# Developing a data-driven algorithm for guiding selection between cognitive behavioral therapy, fluoxetine, and combination treatment for adolescent depression

**DOI:** 10.1038/s41398-020-01005-y

**Published:** 2020-09-21

**Authors:** Meredith Gunlicks-Stoessel, Bonnie Klimes-Dougan, Adrienne VanZomeren, Sisi Ma

**Affiliations:** 1 Department of Psychiatry, University of Minnesota, Minneapolis, MN USA; 2 Department of Psychology, University of Minnesota, Minneapolis, MN USA; 3 Institute of Health Informatics, School of Medicine, University of Minnesota, Minneapolis, MN USA

**Keywords:** Depression, Psychology

## Abstract

Treating adolescent depression effectively requires providing interventions that are optimally suited to patients’ individual characteristics and needs. Therefore, we aim to develop an algorithm that matches patients with optimal treatment among cognitive-behavioral therapy (CBT), fluoxetine (FLX), and combination treatment (COMB). We leveraged data from a completed clinical trial, the Treatment for adolescents with depression study, where a wide range of demographic, clinical, and psychosocial measures were collected from adolescents diagnosed with major depressive disorder prior to treatment. Machine-learning techniques were employed to derive a model that predicts treatment response (week 12 children’s depression rating scale-revised [CDRS-R]) to CBT, FLX, and COMB. The resulting model successfully identified subgroups of patients that respond preferentially to specific types of treatment. Specifically, our model identified a subgroup of patients (25%) that achieved on average a 16.9 point benefit on the CDRS-R from FLX compared to CBT. The model also identified a subgroup of patients (50%) that achieved an average benefit up to 19.0 points from COMB compared to CBT. Physical illness and disability were identified as overall predictors of response to treatment, regardless of treatment type, whereas baseline CDRS-R, psychosomatic symptoms, school missed, view of self, treatment expectations, and attention problems determined the patients’ response to specific treatments. The model developed in this study provides a critical starting point for personalized treatment planning for adolescent depression.

## Introduction

Depression during adolescence is a significant public health problem that is continuing to rise, with a 12-month prevalence of 8.7% in 2005 compared to 11.3% in 2014^1^. Adolescent depression increases the risk of death by suicide and can greatly interfere with the achievement of critical developmental tasks, with deficits observed in academic, social, emotional, and neurobiological functioning^2–4^. Without proper treatment, adolescent depression also carries a risk for continued impairment and long-lasting impacts on physical and mental health throughout the lifespan^5,6^. As such, identifying the most effective treatments for adolescent depression is critical to promoting healthy functioning during this formative developmental stage and beyond.

While several evidence-based treatments (EBTs) have well-established empirical support, including psychotherapies, medications, and their combination, the overall response rates are alarmingly low; 30–50% of adolescents do not respond^7,8^. Ineffective treatment discourages many patients from pursuing further treatment^9^ and unnecessarily exposes patients to medication side effects, undue expenses on health care services, and lost work and/or school time^10^. Because depression is a heterogeneous disorder with multiple etiologies and symptom profiles, a lack of attention to individual characteristics when selecting among EBTs may explain the modest response rates. Unfortunately, treatment providers have little available information to guide the selection between EBTs for a particular patient, obliging them to rely on clinical judgement, which can be variably and erroneously influenced by clinician-specific factors^11^. Guidelines, or algorithms, that optimally match treatments to patients’ individual characteristics, needs, and circumstances are needed^12^. The primary aim of the current study was to develop an algorithm to guide treatment selection for adolescents with depression.

Developing algorithms for treatment matching requires identifying and characterizing subgroups of adolescents who are expected to respond differently to different treatments. Empirical efforts to develop algorithms have typically involved using data from clinical trials to search for constructs that can be identified prior to treatment that predict or moderate treatment outcome^13,14^. Unfortunately, this research has produced little that is of practical utility for adolescents. Many of the variables examined have served to predict prognosis with any treatment, as opposed to moderators, which differentiate who would respond to one treatment as compared to another^15^. Thus, these studies provide little insight into which treatment is best suited for a specific patient. Other studies have identified patient characteristics that moderate treatment outcome, including abuse history, family dynamics, the severity of depression, annual income, and comorbid diagnoses^15–17^. However, these studies evaluated single risk factors in isolation (e.g., they examined only abuse history as a moderator or only family dynamics as a moderator). This approach provides information regarding treatment effect moderation, but it has limited applicability in the clinical setting. First, given that the etiology, course, and clinical expression of depression is complex, individual moderators likely only provide partial information regarding an individual’s response to treatment. Second, it is likely that the moderators identified in isolation would provide conflicting treatment effect estimation for the same patient^18^. For example, for the same patient, their abuse history may indicate that they will have a better response to one treatment, but their family dynamics may indicate that another treatment is more suitable.

Therefore, to develop an effective algorithm to guide treatment selection, the first challenge is to examine a large collection of variables as potential predictors and moderators and model their combined effect on the outcome of interest. This task is not trivial. Given a large number of candidate variables in a data set with a relatively small sample size, searching for significant predictors and moderators naively and exhaustively will likely result in the identification of false-positive predictors and an overfitted model. To avoid these issues, we employed modern machine-learning methods and protocols. Specifically, we used the generalized local learning (GLL) algorithm^19,20^ for feature selection. The GLL belongs to the class of causal feature selection algorithms where the data generation process is estimated and leveraged to aid feature selection. It employs a search strategy that traverses a constrained search space to minimize false discovery and uses conditional independence tests to derive a minimal set of variables that contains the maximum amount of information regarding the outcome given all collected variables. This method has demonstrated success in predictive modeling in various domains^19,20^. In addition, we also applied the cross-validation protocol, such that model performance estimation is conducted on samples that were not examined during model construction. This protocol results in unbiased performance estimation^21,22^. These machine-learning methods and protocols have recently been utilized to successfully construct high-quality models using data sets with modest sample sizes for diagnosis and prognosis using observational data^23–25^ and for treatment assignment using experimental data^26,27^ in mental health and other fields of medicine.

One other challenge in developing effective treatment selection algorithms is the ability to estimate the treatment effects correctly. There are many statistical techniques for adjusting for biases when estimating treatment effects from data where treatments are not randomly assigned. However, these methods leave open the possibility of residual confounding and are subject to undetectable latent confounding. In contrast, treatment effect estimation from randomized clinical trials eliminates confounding both from measured and latent variables. Therefore, we chose to leverage the only large clinical trial with an extensive baseline assessment battery that compares the three primary treatments for adolescent depression: The treatment of adolescents with depression study (TADS)^7^. TADS was a large, multi-site clinical trial of cognitive-behavioral therapy (CBT), fluoxetine (FLX), combination treatment (COMB), and placebo (PBO).

We hypothesized that by applying state-of-the-art machine-learning methods to data from TADS, we could develop a treatment selection algorithm that could successfully identify subpopulations of patients that benefit from FLX vs. COMB vs. CBT.

## Method

### Participants

Participants were adolescents (age 12–17) with a primary DSM-IV diagnosis of current major depressive disorder (MDD) who participated in TADS. The study design and methods have been described in the previous papers^7,28,29^. For the current study, because our aim was to predict treatment response during the acute treatment phase, we included participants who completed assessments at the end of the acute phase (week 12), and did not include participants in PBO. This resulted in 282 patients. The average age was 14.7 years (SD = 1.5) and the proportion of males was 42.2%. The ethnic composition of the sample was 75.9% White/Caucasian, 9.9% African American/Black, 2.1% Hispanic and Black, 7.4% Hispanic and White, 1.1% Asian, 0.4% Pacific Islander. Participants who did and did not have week 12 data were similar in demographic and clinical characteristics, with the exception of being significantly more likely to be male (χ^2^(1)= 5.50, *p* = 0.02). Written informed consent and assent were obtained from at least one parent/caregiver and the adolescent. The coordinating center at Duke University Medical Center and the Institutional Review Board at each site approved and monitored the study. The Data and Safety Monitoring Board of the National Institute of Mental Health also performed quarterly reviews.

The PBO condition of the TADS study was not included in our study, as our aim was to develop models that predicted differential treatment responses among treatments that could be expected to be delivered in clinical settings.

### Procedures

Adolescents enrolled in the study were randomly assigned to one of the four acute treatment conditions: CBT, FLX, COMB, or PBO. Assessments were conducted at baseline, week 6, and week 12 during the acute treatment phase. Clinical assessments were completed by an independent evaluator who was blind to treatment condition. Adolescents and parents also completed self-report measures.

### Baseline measures

The baseline measures selected as candidate predictor variables assessed a broad range of variables (184 total) that are risk factors for depression in adolescents, including traumatic life events, maladaptive cognitive styles, dysfunctional/conflictual family environments, and negative treatment expectations. A list of each of the measures included in the predictive model is provided in Table 1. The complete list of all 184 variables, which includes the subscales of each measure, is provided in Supplementary Table 1.

**Table 1:** Baseline measures included in the prognostic model.

Demographics
Demographics questionnaire^29^
Adolescent psychiatric symptoms
Schedule for Affective disorders & schizophrenia for school-age children (K-SADS-PL)^40^
About my life (SIQ JR)^41^
Reynolds adolescent depression scale (RADS)^42^
Brief symptom inventory (BSI)^43^
Conners–Wells adolescent self-report scale (CASS)^36^
Conners parent ratings scale (CPRS)^34^
Health of the nation outcome scale for children and adolescents (HoNOSCA)^44^
Multidimensional anxiety scale for children (MASC)^45^
Personal experience screening questionnaire (PESQ)^46^
Health and development
Female menstrual cycle (FMC)^47^
Physical symptoms checklist (PSC)^48^
Tanner staging form (TSF)^49,50^
Wechsler intelligence scale for children (WECH)^51^
Family functioning
Conflict behavior questionnaire (CBQ) adolescent report on mother^52^
Conflict behavior questionnaire (CBQ) adolescent report on father^52^
Conflict behavior questionnaire (CBQ) parent report^52^
Dyadic adjustment scale (DAS)^53^
Family assessment measure (FAM)^54^
Issues checklist adolescent report (ICA)^55^
Issues checklist parent report (ICAP)^55^
School functioning
School functioning questionnaire^29^
General psychosocial functioning
Children’s global assessment scale (CGAS)^56^
Pediatric life events screen (PLES)^57^
Pediatric quality of life scale (PQLQ)^57^
Life events
Teen trauma history (TRAUMA)^17^
Cognitive style
Beck hopelessness scale (BHS)^58^
Modified children’s attributional style questionnaire (CASQ)^59^
Children’s negative cognitive error questionnaire (CNCE)^60^
Cognitive triad inventory for children (CTI)^35^
Dysfunctional attitudes scale (DAS)^61^
Social problem-solving inventory – revised (SPSI)^62^
Attitudes toward treatment
Stages of change (SOC)^63^
Treatment expectancy adolescent report^15^
Treatment expectancy parent report^15^
Treatment history
Child and adolescent services assessment (CASA)^64^
Parent psychiatric symptoms
Beck depression inventory (BDI)^65^
Conners’ adult ADHD rating scale (CAARS)^66^

### Outcome measure

The children’s depression rating scale-revised (CDRS-R)^30^ is a well-validated, clinician-administered, semi-structured interview that assesses symptoms of depression experienced during the previous two weeks. While originally developed for use with children, the measure is also widely used with adolescents and has demonstrated good reliability and validity with this age group^31^. Symptoms assessed are aligned, but do not completely overlap, with DSM-IV^32^ criteria for depressive disorders (e.g., sadness, irritability, anhedonia, appetite, self-esteem, guilt, suicidality). Parents and teens are separately interviewed, and their individual responses are scored. Interviewers (i.e., independent evaluators blind to treatment condition) later integrate parent and teen’s individual responses into one consensus summary score for each item, which are then summed to produce an overall raw summary score. Raw scores between 30 and 43 indicate moderate concerns, while raw scores above 44 indicate greater severity and suggest a higher probability of a confirmed depression diagnosis. In the current study, CDRS-R scores at week 12 were the primary outcome measure. The intraclass correlation coefficient for the CDRS-R total score at baseline was 0.95, suggesting excellent interrater reliability^7^.

### Predictive modeling and statistical analysis

#### Overall strategy

Our goal was to build a model that predicts participants’ treatment response (week 12 CDRS-R) to CBT, FLX, and COMB treatment is given their baseline characteristics. This model can be used to predict a specific participant’s week 12 CDRS-R score if they were treated with CBT, FLX, or COMB. The treatment that corresponds to the best (statistically significant) model-predicted week 12 CDRS-R score is then deemed the best treatment for that participant.

On a high level, the predictive model for week 12 CDRS-R could contain two types of independent variables. The first type is the predictors of outcome or the main effects; these variables’ relationships with the outcome do not change across different treatment conditions. The second type is the moderators of the treatment; these variables exhibit significant interaction with the treatment and influence the effect of the treatments. The existence of moderators is critical for personalized treatment assignment since all participants would show a similar treatment effect when the same treatment is given in the absence of moderation. Noting that, the predictor variables are technically not necessary for determining which treatment is more effective for a specific participant (more about this in the result section); however, these variables will improve the accuracy of the prediction for week 12 CDRS-R.

To achieve better model generalizability and interpretability, we employed variable selection. To obtain unbiased performance estimation, we used the leave-one-out cross-validation procedure. For a more detailed description, see the sections below. The design of the overall analytical protocol follows the general recommendation of using complex machine-learning methods for differential treatment effect modeling described in a recent review ^18^.

### Variable selection and model construction

**Fig. 1:**
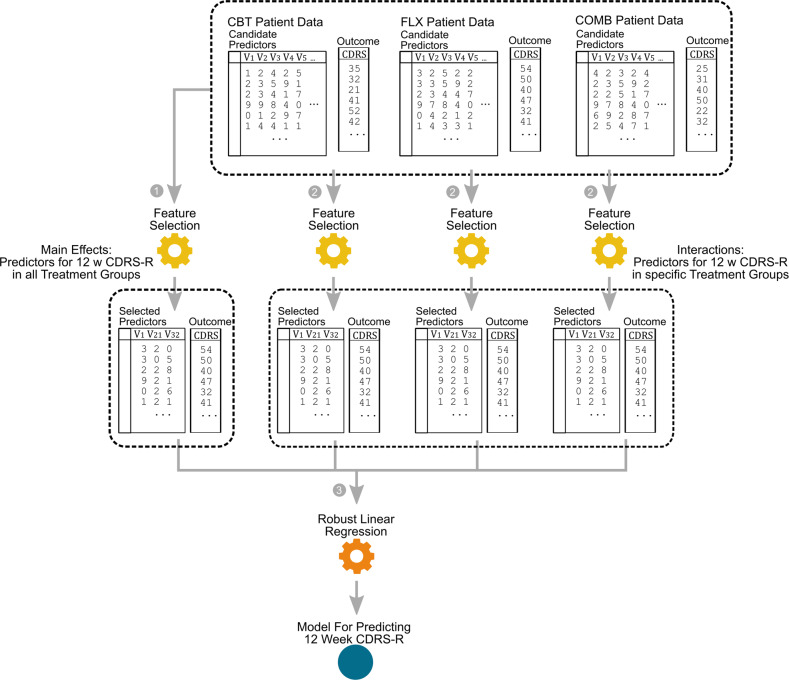
**Illustration of analytical strategy.** Step 1: To identify the predictors or the main effect of the outcome, we used data from all participants regardless of what treatment they received. Step 2: To identify the moderators of different treatments, we used data from participants that were given that treatment. The feature selection method generalized local learning (GLL) was employed to avoid overfitting. Step 3: The predictive model for week 12 CDRS-R were constructed using a robust linear regression based on the identified variables from the previous step. Specifically, the variables identified in step 1 (treatment effect predictors) were built into the regression as main effects, and the variables identified in step 2 (moderators) were built into the regression as interaction effects with their corresponding treatment.

The first step in constructing the predictive model is to identify the predictors and the moderators of the outcome. To identify the predictors or the main effect of the outcome, we used data from all participants regardless of what treatment they received (Fig. 1, step 1). To identify the moderators of different treatments, we used data from participants that were given that treatment (Fig. 1, step 2). Given a large number of baseline characteristics and the relatively small sample size, searching for significant predictors and moderators naively and exhaustively will likely result in false discovery and overfitting. Therefore, we employed a state-of-the-art machine-learning algorithm, the generalized local learning (GLL) algorithm^19,20^ for feature selection.

The predictive model for week 12 CDRS-R was constructed using a robust linear regression based on the identified variables from the previous step (Fig. 1, step 3). Specifically, the variables identified in step 1 (treatment effect predictors) were built into the regression as main effects, and the variables identified in step 2 (moderators) were built into the regression as interaction effects with their corresponding treatment.

### Predicting CDRS-R outcome

Given the derived predictive model and characteristics of the individual in question, we computed the model-predicted week 12 CDRS-R score for a specific treatment option by plugging in the values of baseline characteristics and the treatment in question into the predictive model. We illustrate this with predicting week 12 CDRS-R using the model in Table 2, i.e.: $$\begin{array}{l}12\,{\mathrm{weeks}}\,{\mathrm{CDRSR}} = - 11.03 + 54.52 \times {\mathrm{FLX}} + 26.77 \times {\mathrm{COMB}} + 3.08 \times {\mathrm{physical}}\,{\mathrm{illness}} + 0 \times CDRSR\\ + 0.72 \times {\mathrm{CBT}} \times {\mathrm{CDRSR}} + 0.72 \times {\mathrm{CBT}} \times {\mathrm{Som}}\,{\mathrm{Sxs}} + \,0.5 \times {\mathrm{CBT}} \times {\mathrm{school}}\,{\mathrm{missed}} - 0.63 \times {\mathrm{FLX}} \times {\mathrm{view}}\,{\mathrm{of}}\,{\mathrm{self}}\\\, + \,4.46 \times {\mathrm{COMB}} \times {\mathrm{Tx}}\,{\mathrm{expectation}} + 0.45 \times {\mathrm{COMB}} \times {\mathrm{attn}}\,{\mathrm{probs}}\end{array}$$. Consider an individual with the following baseline characteristics: physical illness and disability score = 4, baseline CDRS-R = 70, psychosomatic symptoms = 6, number of school days missed = 12, view of self score = 3, treatment expectation = 2, attention problems = 15. To predict this individual’s week 12 CDRS-R score given a particular treatment, we plug in a value of 1 for the treatment in question and plug in a value of 0 for the other treatments. The model-predicted CDRS-R at week 12, when treated with FLX, is: $$\begin{array}{l} - 11.03 + 54.52 \times 1 + 26.77 \times 0 + 3.08 \times 4 + 0 \times 70 + 0.72 \times 0 \times 70 + 0.72 \times 0 \times 6\\ + 0.5 \times 0 \times 12 - 0.63 \times 1 \times 3 + 4.46 \times 0 \times 2 + 0.45 \times 0 \times 15 = 53.9\end{array}$$; the model-predicted CDRS-R at week 12 when treated with CBT is: $$\begin{array}{l} - 11.03 + 54.52 \times 0 + 26.77 \times 0 + 3.08 \times 4 + 0 \times 70 + 0.72 \times 1 \times 70 + 0.72 \times 1 \times 6\\ + 0.5 \times 1 \times 12 - 0.63 \times 0 \times 3 + 4.46 \times 0 \times 2 + 0.45 \times 0 \times 15 = 61.9\end{array}$$; the model-predicted CDRS-R at week 12 when treated with COMB is: $$\begin{array}{l} - 11.03 + 54.52 \times 0 + 26.77 \times 1 + 3.08 \times 4 + 0 \times 70 + 0.72 \times 0 \times 70 + 0.72 \times 0 \times 6\\ + 0.5 \times 0 \times 12 - 0.63 \times 0 \times 3 + 4.46 \times 1 \times 2 + 0.45 \times 1 \times 15 = 43.7\end{array}$$. The model-predicted differential treatment response between a pair of treatments is the predicted week 12 CDRS-R of one treatment minus that of the other treatment, e.g., the treatment difference between CBT and FLX for the individual above is $61.9 - 53.9 = 8.0$. The individual’s predicted benefit of treating with FLX as compared to CBT is 8 CDRS-R points.

**Table 2:** Model for predicting week 12 CDRS-R given baseline characteristics and treatment.

	Coefficient	SE	95% CI		*t*	*p*
Intercept	−11.03	8.26	−27.22	5.16	−1.33	0.18304
FLX	54.52	10.48	33.99	75.06	5.20	3.84E−07
COMB	26.77	9.83	7.49	46.04	2.72	0.006917
Physical illness	3.08	1.01	1.09	5.07	3.04	0.002606
CDRS-R	0.00	0.08	−0.16	0.15	−0.01	0.9886
CBT × CDRS-R	0.72	0.16	0.40	1.04	4.41	1.48E−05
CBT × Som Sxs	0.72	0.27	0.20	1.24	2.73	0.006721
CBT × school missed	0.50	0.24	0.02	0.97	2.06	0.039952
FLX × view of self	−0.63	0.25	−1.11	−0.14	−2.54	0.011622
COMB × Tx expectation	4.46	1.05	2.40	6.52	4.24	3.02E−05
COMB × attn probs	0.45	0.16	0.14	0.77	2.80	0.005522

*CBT* cognitive behavioral therapy, *FLX* fluoxetine, *COMB* combination treatment; *physical illness* health of the nation outcome scales (HoNOS) physical illness or disability problems subscale; *CDRS-R* baseline children’s depression rating scale-revised (CDRS-R) score; *Som Sxs* psychosomatic subscale of the Conners parent ratings scale (CPRS); *school missed* number of missed school days in the last two month; *view of self* Cognitive triad inventory for children (CTI) view of self subscale; *Tx expectation* adolescents’ expectation of treatment response with the COMB treatment; *attn probs* baseline cognitive problems/inattention subscale of the Conners–Wells adolescent self-report scale (CASS).

### Performance estimation

To obtain unbiased performance estimation, we employed the leave-one-out cross-validation procedure^21,22^. This procedure ensures that performance estimations are obtained from participants that were not used during the model construction. More specifically, we conducted a feature selection and trained the predictive model based on all but one participant. The participant that was not part of the model training process was reserved for performance estimation and is referred to as the testing participant. The rest of the participants from which the models were built are referred to as the training participants. We predicted the treatment response of the testing participant given the three different treatments by applying the model derived from the training participant. We also computed the predicted benefit for each pair of treatments. The above process was repeated with every patient serving as the testing participant once.

To evaluate the utility of our model for treatment assignment, we stratified the participants by their predicted benefit into four strata corresponding to quartiles. The participants that belong to a stratum that corresponds to a higher quartile were expected to achieve more actual benefit compared to the participants that were assigned to a lower quartile. To assess the average actual benefit of participants receiving one treatment vs. another within a stratum, we compared the mean week 12 CDRS-R of the participants that received one treatment vs. the other treatment. We used the student *t*-test to determine if significant treatment benefit was achieved. *p*-values were false discovery rate (FDR) adjusted for multiple comparisons^33^. To evaluate the robustness of our analysis, we also used 20 fold cross-validation as an alternative performance estimation protocol (Supplementary Table 1). In addition, we also conducted two permutation tests to examine the robustness of the overall analytical protocol and the feature selection method (Supplementary Tables 2 and 3).

## Results

### Predicting week 12 CDRS-R using baseline characteristics

**Fig. 2:**
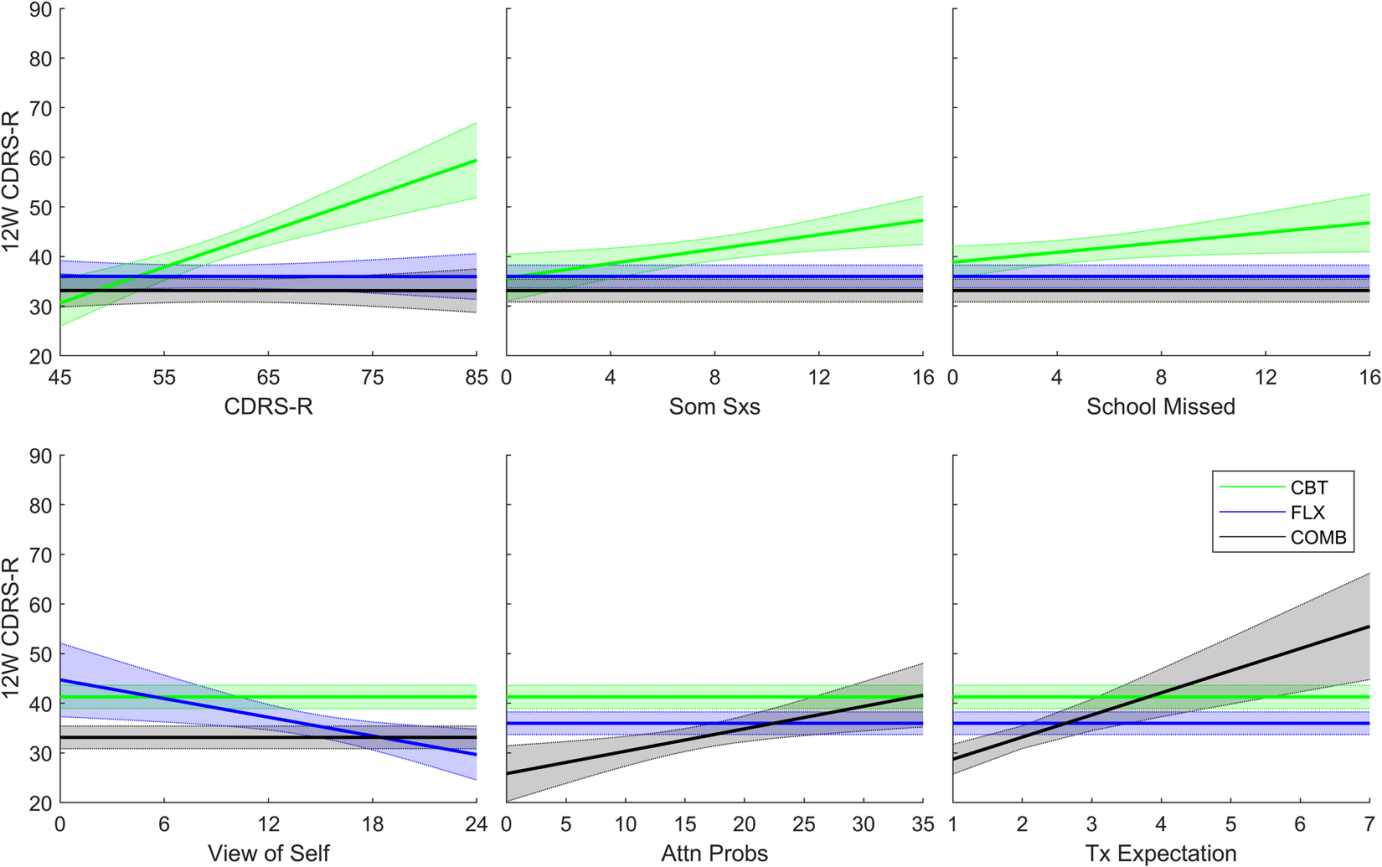
Plots of treatment moderators (interactions) from the final model. Treatment moderators and their effect of week 12 CDRS-R are visualized. The positive slopes of CDRS-R, Som Sxs, and School Missed for CBT indicate that the higher the value of these variables, the higher the week 12 CDRS-R if the patients were treated with CBT. The value of these variables do not influence the treatment effects for FLX and COMB, as indicated by the zero slopes; the Negative slopes of view of self for FLX indicate that the higher the view of self, the lower the week 12 CDRS-R if the patients were treated with FLX. The positive slopes of Attn Probs and Tx Expectation for COMB indicate that the higher values of these variables, the higher the week 12 CDRS-R if the patients were treated with COMB (higher values of Tx Expectation represent low treatment expectation). Shading around the slopes represents 95% predictive intervals. See Table 2 for abbreviations of variable names.

Table 2 shows the model for predicting week 12 CDRS-R given baseline characteristics and treatment. The baseline characteristics that are predictors of week 12 CDRS-R (significant main effects) are physical illness and disability problems (the health of the nation outcome scales (HoNOS) physical illness or disability problems subscale). The effects of the moderators on week 12 CDRS-R are shown in Table 2 and visualized in Fig. 2. Specifically, the characteristics that moderate CBT treatment response is baseline CDRS-R, psychosomatic symptoms (psychosomatic subscale of the Conners parent ratings scale (CPRS))^34^, and the number of school days missed. Higher baseline CDRS-R, more severe psychosomatic symptoms, and more missed school days are associated with a worse week 12 CDRS-R score when treated with CBT. The baseline characteristic that moderates response to FLX treatment is the cognitive triad inventory for children (CTI)^35^ view of the self subscale. A more positive view of self is associated with better response to FLX treatment. The baseline characteristics that moderate response to the COMB treatment are baseline cognitive problems/inattention subscale of the Conners–Wells adolescent self-report scale (CASS)^36^ and adolescents’ expectation of treatment response with the COMB treatment. More severe attention problems and lower treatment expectations are indicative of worse treatment response to COMB treatment.

### Treatment selection

To evaluate the utility of our model for treatment assignment, we stratified the participants by their predicted benefit from one treatment vs. another into four strata corresponding to quartiles. We then compared the mean week 12 CDRS-R of the participants that received one treatment vs. the other treatment to assess the average actual benefit of participants receiving one treatment vs. another within a stratum. The above procedure was embedded in a leave-one-out cross-validation protocol. This result is shown in Table 3 and Supplementary Fig. 1. Regarding the model’s effectiveness for treatment selection between CBT vs. FLX, the patients that belong to the stratum with the highest model-predicted benefit for FLX exhibit significant benefit (adjusted *p* = 0.001) from FLX as compared to CBT, with an average benefit of 16.9 CDRS-R points. The patients that belong to the other three strata did not show significant benefit from FLX compared to CBT. This indicates that our model is successful in identifying a subgroup of patients that respond better to FLX vs. CBT. Regarding the model’s effectiveness for treatment selection between CBT vs. COMB, the patients that belong to the top two strata with higher predicted benefit for COMB significantly benefitted from COMB compared to CBT. Specifically, the patients in the top stratum (75–100%) showed 19.0 (adjusted *p* = 0.002) CDRS-R points benefit. The patients in the next stratum (50–75%) showed 8.4 (adjusted *p* = 0.003) CDRS-R points benefit. The patients in the bottom two strata did not show significant benefit from COMB compared to CBT. This indicates that our model is successful in identifying a subgroup of patients that respond better to COMB vs. CBT. Finally, regarding the model’s effectiveness for treatment selection between FLX vs. COMB, patients in all strata did not show significant benefit from COMB compared to FLX or vice versa, i.e., the model failed to identify patients that responded differently with FLX vs. COMB.

**Table 3:** Patient CDRS-R benefits stratified by model prediction.

CBT vs. FLX										
Predicted benefit strata	Treated with CBT			Treated with FLX			Estimated benefit			
	*M*	SD	*N*	*M*	SD	*N*	*M*	Cohen’s *D*	*p*	Adj. *p*
0–25%	35.1	11.0	22	34.0	12.3	28	1.1	0.1	0.74	0.74
25–50%	35.5	8.6	20	36.7	10.8	23	−1.2	−0.1	0.68	0.74
50–75%	39.4	11.1	27	37.6	16.4	21	1.8	0.1	0.66	0.74
75–100%	**56.2**	**15.1**	**21**	**39.3**	**11.2**	**25**	**16.9**	**0.9**	**0.00**	**0.00**

CBT vs. COMB										
Predicted benefit strata	Treated with CBT			Treated with COMB			Estimated benefit			
	*M*	SD	*N*	*M*	SD	*N*	*M*	Cohen’s *D*	*p*	Adj. *p*
0–25%	35.8	11.3	20	30.7	9.9	21	5.0	0.3	0.14	0.41
25–50%	36.9	11.1	22	34.8	13.1	24	2.1	0.1	0.57	0.74
50–75%	**39.3**	**9.2**	**29**	**30.8**	**7.9**	**24**	**8.4**	**0.7**	**0.00**	**0.00**
75–100%	**55.8**	**17.4**	**19**	**36.8**	**14.6**	**26**	**19.0**	**0.8**	**0.00**	**0.00**

FLX vs. COMB										
Predicted benefit strata	Treated with FLX			Treated with COMB			Estimated benefit			
	*M*	SD	*N*	*M*	SD	*N*	*M*	Cohen’s *D*	*p*	Adj. *p*
0–25%	39.2	13.8	25	35.6	11.2	18	3.6	0.2	0.37	0.65
25–50%	34.5	9.2	30	33.0	9.9	24	1.6	0.1	0.55	0.74
50–75%	36.1	13.0	26	32.0	10.8	28	4.1	0.2	0.21	0.50
75–100%	38.4	16.1	16	34.0	15.2	25	4.4	0.2	0.38	0.65

Bold indicates a significant treatment benefit in predicted benefit strata.

*CDRS-R* children’s depression rating scale-revised, *CBT* cognitive behavioral therapy, *FLX* fluoxetine, *COMB* combination treatment.

*CBT vs. FLX:* Rows represent groups of patients that are predicted to benefit from FLX over CBT with different magnitudes (bottom 25%, 25–50%, 50–75%, top 25%). The estimated benefit from FLX compared to CBT within each stratum is computed as the difference in CDRS-R between the patients who were treated with CBT and those treated with FLX. The participants who were predicted to benefit the most (top 25%) were estimated to benefit significantly from FLX with on average 16.9 CDRS-R difference. Adj *p* = adjusted *p*-value.

*CBT vs. COMB:* Rows represent groups of patients that are predicted to benefit from COMB over CBT with different magnitudes (bottom 25%, 25–50%, 50–75%, top 25%). The estimated benefit from COMB compared to CBT within each group is computed as the difference in CDRS-R between the patients who were treated with CBT and those treated with COMB. The participants who were predicted to benefit more from COMB (top 50%) were estimated to benefit significantly from COMB. Adj *p* = adjusted *p-*value.

*FLX vs. COMB:* Rows represent groups of patients that are predicted to benefit from COMB over FLX with different magnitudes (bottom 25%, 25–50%, 50–75%, top 25%). The estimated benefit from COMB compared to FLX within each group is computed as the difference in CDRS-R between the patients who were treated with FLX and those treated with COMB. Adj *p* = adjusted *p-*value.

## Discussion

Treating depression more effectively requires providing patients with treatment that is optimally matched to their individual characteristics, needs, and circumstances. In this study, machine-learning techniques were applied to a wide range of demographic, clinical, and psychosocial data collected in TADS to build a model to predict overall treatment response after 12 weeks. This model also successfully stratified patients according to their responsiveness to FLX vs. COMB vs. CBT. The findings are promising and provide preliminary evidence that algorithms can be developed from parent and youth self-report measures that can be easily administered in clinic settings. The methodology used in this study can also be applied broadly to data from other clinical trials for depression, other psychiatric disorders, and other domains of medicine.

Our model identified treatment effect predictors and moderators for CBT, FLX, and COMB. Regardless of treatment, adolescents with less severe physical illness and disability problems reported better outcomes. When considering the results that may guide treatment assignment, for CBT, fewer endorsed depressive and somatic symptoms and fewer missed school days at baseline were associated with a better treatment outcome. For FLX, adolescents who reported more favorable views of themselves at baseline reported fewer depression symptoms post-treatment. Adolescents demonstrated a greater response to COMB if they reported fewer problems with inattention at baseline and had more favorable expectations regarding the effectiveness of COMB.

Our method is data rather than hypothesis-driven and thus may identify predictors or moderators that are novel and not part of the current clinical perceptions of what constructs ought to be critical drivers of treatment decision making. Nevertheless, some of the treatment response predictors and moderators identified by our algorithms align in some respects with previously-identified predictor and/or moderator variables in TADS, such as depression severity, attention problems, and treatment expectancy^15,37^. These constructs are likely to represent potential treatment mechanisms. However, others are new, including psychosomatic symptoms, physical illness, and disability problems, view of self, and a number of missed days of school. It is not yet clear at face value why these variables are important. However, considering what these different treatments do and do not aim to target, some of these newly identified empirically generated variables are consistent with theory. CBT’s purported mechanisms of action are correcting depression-related cognitive distortions and increasing engagement in pleasurable activities. Thus, it makes sense that adolescents with higher levels of somatic symptoms may not respond as well to CBT, since CBT does not aim to directly remediate somatic symptoms. Similarly, adolescents who reported negative views of themselves might not be expected to respond more favorably to FLX, as FLX does not aim to target negative cognitions. Adolescents with attention difficulties may have benefited more from COMB because the medication enabled these adolescents to more easily focus and engage in the cognitive work of CBT.

Some variables identified as moderators in other studies were not represented in our final model, including abuse history, family dynamics, annual income, and comorbid diagnoses. Other variables that one might expect to see from a theoretical standpoint also were not identified (e.g., none of the cognitive variables predicted outcome with CBT). This may be due to differences in the study population (e.g., depression severity, inclusion/exclusion criteria) and/or analytic approach (previous studies examined risk factors in isolation and not in combination, as we did in our study). Moreover, while we chose not to include the PBO patients since the goal of this study was to determine the best clinically available treatment for a given patient, several previous studies identified moderators with patients from the PBO group. Finally, it is worth noting that some previously reported predictors and moderators were not part of our model since they are not conditionally independent of the 12 weeks CDRS-R given the variables that are in our model. That is, they do not provide additional information regarding 12 weeks CDRS-R after considering the predictors and moderators identified in the current study (more details are shown in Supplementary Table 4).

Our model demonstrated the ability to stratify patients according to their differential response to treatment using their baseline characteristics. It is worth noting that, although only subpopulations of patients exhibit predictable differential treatment response, the ability to identify these sub-populations benefits all patients since the patients who are not likely to benefit from FLX or COMB over CBT could choose the treatment that best suits their preference and circumstances. More specifically, to translate our model (FLX vs. CBT, COMB vs. CBT) into an implementable algorithm, we would suggest starting by considering the model with the least burdensome treatment with regard to time, cost, side effect profile, and use of resources. The hope is that this approach will maximize clinical benefits, minimize burdens, and lead to more efficient and cost-effective treatment. As such, one approach (Supplementary Fig. 2) would be to assume that FLX would be less burdensome than COMB and therefore would be recommended first. That is, a provider would first determine if a depressed adolescent fits into the profile of those who are likely to benefit from FLX over CBT, and, if so, FLX would be recommended. If the patient does not fit the FLX over CBT profile, it would then be determined if his/her profile is consistent with those who are likely to benefit from COMB over CBT. If so, COMB would be recommended. For the remaining patients, FLX, CBT, and COMB would be expected to be equally effective, providing the opportunity for patient preference. In practice, the treatment provider would administer a baseline battery of relevant measures included in the model (Table 2) and enter the scores (an example computation shown in the methods section) into the decision support algorithm (e.g., Supplementary Fig. 2). The algorithm would then estimate the treatment response given different treatments. The results of the algorithm, in conjunction with patient preferences, would be used to determine which treatment would be initiated for the adolescent.

The potential benefits of algorithm assignment-guided decision making are likely to significantly impact the course and outcome of treatment in adolescents. To place the results of this study in the context of the original acute-phase outcomes reported in TADS (2004), in the original study, the mean difference in week 12 CDRS-R score between CBT and FLX for all patients who received them was 5.8 points. In our reanalysis, the mean difference between CBT and FLX for the subgroup that benefitted more from FLX than from CBT was 16.9. For the remaining patients, CBT and FLX would be expected to be equally effective. The mean difference in week 12 CDRS-R score between COMB and CBT for all patients who received them was 8.3. In our reanalysis, the mean difference between COMB and CBT for the subgroup that benefitted more from COMB than from CBT was 19.0. For the remaining patients, CBT and COMB would be expected to be equally effective. By pooling the combined effect of unique baseline variables in our machine-learning approach, we were able to add personalized prediction of treatment benefit.

This foundational research provides a starting point for developing a clinical decision support system for treatment response. The next step would be to validate and apply these models in clinical settings for treatment selection. An algorithm-based approach to personalized treatment that attends to characteristics of the individual patient, as opposed to DSM-V symptoms and diagnostic criteria alone, represents both a significant advance and a departure from usual clinical practice. With recent innovations in technology, such algorithms can now be integrated into clinical care via interfacing with patients’ electronic medical records, thus creating an automated and user-friendly method of personalized treatment planning. Because it remains unclear to what extent clinicians will embrace these ideas, future research will be necessary to not only assess the effectiveness, but also the feasibility and acceptability of the clinical decision support system, as well as the facilitators and barriers to successful deployment. An important future direction for research will be to investigate the statistical model in real-life clinical practice settings in which patients and providers collaborate to select among treatment options.

There are also a number of additional next steps with respect to improving the current model and thus more accurately assigning patients to suitable treatment, including considering (a) a broader array of variables as predictors or moderators (e.g., multiple levels of analysis including behavioral and biological candidates^38,39^); (b) a broader array of treatments, (c) a more diverse population of adolescents with depressive symptoms to enhance external validity (e.g., mild to treatment-resistant depression, samples with greater diversity with regard to race, ethnicity, and socio-economic status) and (d) longer outcome windows.

Some fields have made rapid progress toward personalization (e.g., cancer research), while these approaches are just beginning to be explored for mental illness. Although this line of work requires a considerable investment, the individual and societal cost of inability to match individuals with the best treatment is substantial. The promise of personalization is that favorable treatment outcomes will be achieved with as little treatment as possible, which maximizes clinical benefits, minimizes burdens, and leads to more efficient and cost-effective service delivery. The current study provides the first machine-learning-based algorithms that direct selection among the most widely disseminated evidence-based treatments for depression in adolescents. Algorithms like these have the potential to innovate clinical practice and improve treatment outcomes, particularly when part of a broader approach which considers how best to translate these research-based algorithms into practice.

## Supplementary information

supplemental information

## Data Availability

Codes for all analysis were implemented in Matlab R2018a, with both custom scripts and existing Matlab functions. Custom script was used for the GLL feature selection. Implementation details of the GLL are discussed in Alifaris et al.^19,20^. Matlab fitlm function was used to generate the regression model.
