# Supplementary material for: Developing a data-driven algorithm for guiding selection between cognitive behavioral therapy, fluoxetine, and combination treatment for adolescent depression
Source: Transl Psychiatry. 2020 Sep 21;10:321. doi: 10.1038/s41398-020-01005-y (PMC7506003; doi:10.1038/s41398-020-01005-y)
Supplement: Supplementary file 1 — supplemental information [file 41398_2020_1005_MOESM1_ESM.docx]

**I. 20 fold cross validation**

We conducted 20 fold cross validation as an alternative performance estimation strategy. Similar to table 3 in the main text, the tables below show the effectiveness of our model in stratifying the patients according to their predictive response to pairs of treatments. The estimates from the 20 fold-cross validation is similar to that of the leave-one-out cross validation (main text table 3).

Supplemental Table 1: Patient CDRS-R Benefits Stratified by Model Prediction (estimated using 20 fold cross validation)

CBT vs. FLX

| Predicted Benefit Strata | Treated with CBT | | | Treated with FLX | | | Estimated benefit | | | |
| --- | --- | --- | --- | --- | --- | --- | --- | --- | --- | --- |
|  | *M* | *SD* | *N* | *M* | *SD* | *N* | *M* | *Cohen's D* | *p* | *adj. p* |
| 0~25% | 36.6 | 10.9 | 20 | 33.3 | 12.2 | 28 | 3.3 | 0.2 | 0.34 | 0.58 |
| 25%~50% | 35.5 | 10.0 | 25 | 35.3 | 10.1 | 25 | 0.2 | 0.0 | 0.93 | 0.93 |
| 50%~75% | 39.3 | 12.2 | 28 | 37.6 | 15.0 | 20 | 1.8 | 0.1 | 0.65 | 0.84 |
| **75%~100%** | **59.1** | **12.6** | **17** | **41.9** | **12.7** | **24** | **17.2** | **1.0** | **0.00** | **0.00** |

CBT vs. COMB

| Predicted Benefit Strata | Treated with CBT | | | Treated with COMB | | | Estimated benefit | | | |
| --- | --- | --- | --- | --- | --- | --- | --- | --- | --- | --- |
|  | *M* | *SD* | *N* | *M* | *SD* | *N* | *M* | *Cohen's D* | *p* | *adj. p* |
| 0~25% | 34.3 | 11.1 | 23 | 32.4 | 11.8 | 24 | 2.0 | 0.1 | 0.56 | 0.84 |
| 25%~50% | 38.3 | 10.8 | 23 | 34.7 | 10.0 | 27 | 3.5 | 0.2 | 0.24 | 0.47 |
| **50%~75%** | **39.6** | **10.5** | **24** | **31.6** | **12.0** | **22** | **7.9** | **0.5** | **0.02** | **0.08** |
| **75%~100%** | **55.3** | **16.1** | **20** | **34.8** | **14.2** | **22** | **20.4** | **1.0** | **0.00** | **0.00** |

FLX vs. COMB

| Predicted Benefit Strata | Treated with FLX | | | Treated with COMB | | | Estimated benefit | | | |
| --- | --- | --- | --- | --- | --- | --- | --- | --- | --- | --- |
|  | *M* | *SD* | *N* | *M* | *SD* | *N* | *M* | *Cohen's D* | *p* | *adj. p* |
| 0~25% | 37.5 | 14.5 | 26 | 36.1 | 11.3 | 27 | 1.4 | 0.1 | 0.70 | 0.84 |
| 25%~50% | 37.8 | 11.7 | 29 | 32.7 | 11.9 | 26 | 5.1 | 0.3 | 0.11 | 0.27 |
| 50%~75% | 35.3 | 11.7 | 26 | 29.6 | 9.5 | 21 | 5.7 | 0.4 | 0.08 | 0.23 |
| 75%~100% | 36.1 | 13.9 | 16 | 34.7 | 14.3 | 21 | 1.3 | 0.1 | 0.78 | 0.85 |

Note: CBT = cognitive behavioral therapy, FLX = fluoxetine, COMB = combination treatment.

Bold indicates significant treatment benefit in a predicted benefit strata. CBT vs FLX: Rows represent groups of patients that are predicted to benefit from FLX over CBT with different magnitudes (bottom 25%, 25%-50%, 50-75%, top 25%). The estimated benefit from FLX compared to CBT within each stratum is computed as the difference in CDRS-R between the patients who were treated with CBT and those treated with FLX. The participants who were predicted to benefit the most (top 25%) were estimated to benefit significantly from FLX with on average 16.9 CDRS-R difference. Adj p = adjusted p value.

CBT vs COMB: Rows represent groups of patients that are predicted to benefit from COMB over CBT with different magnitudes (bottom 25%, 25%-50%, 50-75%, top 25%). The estimated benefit from COMB compared to CBT within each group is computed as the difference in CDRS-R between the patients who were treated with CBT and those treated with COMB. The participants who were predicted to benefit more from COMB (top 50%) were estimated to benefit significantly from COMB. Adj p = adjusted p value.

FLX vs COMB: Rows represent groups of patients that are predicted to benefit from COMB over FLX with different magnitudes (bottom 25%, 25%-50%, 50-75%, top 25%). The estimated benefit from COMB compared to FLX within each group is computed as the difference in CDRS-R between the patients who were treated with FLX and those treated with COMB. Adj p = adjusted p value.

**II. Permutation Test**

We conducted two types of permutation analysis to access the robustness of our results.

To evaluate the entire procedure for differential treatment effect modeling, we applied our modeling procedure to a permuted version of the TADS dataset, where the outcome of interest CDRS-R at 12 weeks was permuted. Permuting the CDRS-R removes the differential treatment effect from the dataset while preserving the marginal distribution of the data. We applied our entire modeling procedure to 200 randomly permuted datasets and compared the differential treatment effect obtained from the permuted data to our original results. This procedure generates a null distribution of estimated treatment effects in different predicted benefit strata where a differential treatment effect is not present. Results are summarized in Supplemental table 1: for CBT vs. FLX and CBT vs. COMB, where differential treatment effects were identified from the original TADS data, models built in the permuted datasets do not show increased treatment benefit as the predicted treatment benefit increases, i.e. our procedure does not detect differential treatment effect where there is none. Also, the mean benefit of the original TADS in the 4^th^ strata for both CBT vs. FLX and CBT vs. COMB is greater than the 95^th^ percentile of the permuted datasets. The above indicates that our procedure captures differential treatment effects that exceeds random variation in the data.

To evaluate the feature selection procedure for constructing prognostics models, we generated models with randomly selected features matching the number of features selected by the GLL feature selection. Differential treatment effect was then computed according to models built using these features and evaluated in a similar fashion as the prognostic models generated by GLL. This procedure generates a null distribution of estimated treatment effects in different predicted benefit strata for feature selection. Randomly selected features for prognostic modeling is expected to result in poor prognostic models thus resulting in poor treatment assignment models and no differential treatment effect among different predicted benefit strata. Results are summarized in supplemental table 2: for CBT vs. FLX and CBT vs. COMB, where differential treatment effects were identified by GLL, models built with randomly selected features do not show increased treatment benefit as the predicted treatment benefit increases, i.e. no differential treatment effects were identified when features for prognostics models were randomly selected. Also, the mean benefit of GLL in the 4^th^ strata for both CBT vs. FLX and CBT vs. COMB is greater than the 95^th^ percentile of the random feature selection. The above indicates that the GLL captures differential treatment effect that outperforms randomly selected features.

Supplemental Table 2: Differential treatment benefit derived from original TADS vs. permuted data

CBT vs. FLX

| Predicted Benefit Strata | Original TADS Data | | | Permuted Data | | |
| --- | --- | --- | --- | --- | --- | --- |
|  | treated with CBT | treated with FLX | est. benefit | est. benefit (permuted data) | | |
|  | mean | mean | mean | mean | 5th percentile | 95th percentile |
| 0~25% | 35.14 | 34.04 | 1.10 | 5.65 | -0.95 | 13.43 |
| 25%~50% | 35.45 | 36.70 | -1.25 | 4.30 | -0.92 | 11.48 |
| 50%~75% | 39.37 | 37.62 | 1.75 | 3.28 | -2.59 | 9.27 |
| **75%~100%** | **56.19** | **39.28** | **16.91** | **5.19** | **-0.14** | **11.26** |

CBT vs. COMB

| Predicted Benefit Strata | Orignial TADS Data | | | Permuted Data | | |
| --- | --- | --- | --- | --- | --- | --- |
|  | treated with CBT | treated with COMB | est. benefit | est. benefit (permuted data) | | |
|  | mean | mean | mean | mean | 5th percentile | 95th percentile |
| 0~25% | 35.75 | 30.71 | 5.04 | 8.05 | 1.07 | 15.07 |
| 25%~50% | 36.86 | 34.79 | 2.07 | 7.25 | 0.98 | 12.50 |
| **50%~75%** | **39.28** | **30.83** | **8.44** | **7.61** | **1.64** | **14.13** |
| **75%~100%** | **55.79** | **36.81** | 18.98 | **8.72** | **3.18** | **14.44** |

FLX vs. COMB

| Predicted Benefit Strata | Orignial TADS Data | | | Permuted Data | | |
| --- | --- | --- | --- | --- | --- | --- |
|  | treated with FLX | treated with COMB | est. benefit | est. benefit (permuted data) | | |
|  | mean | mean | mean | mean | 5th percentile | 95th percentile |
| 0~25% | 39.2 | 35.6 | 3.6 | 2.54 | -4.18 | 8.96 |
| 25%~50% | 34.5 | 33.0 | 1.6 | 3.68 | -1.84 | 8.92 |
| 50%~75% | 36.1 | 32.0 | 4.1 | 3.79 | -1.81 | 9.14 |
| 75%~100% | 38.4 | 34.0 | 4.4 | 3.31 | -2.06 | 7.81 |

Supplemental Table 3: GLL feature selection for constructing prognostics models vs. random features.

CBT vs. FLX

| Predicted Benefit Strata | GLL | | | Random Features | | |
| --- | --- | --- | --- | --- | --- | --- |
|  | treated with CBT | treated with FLX | est. benefit | est. benefit (permuted data) | | |
|  | mean | mean | mean | mean | 5th percentile | 95th percentile |
| 0~25% | 35.14 | 34.04 | 1.10 | 4.79 | -1.68 | 11.11 |
| 25%~50% | 35.45 | 36.70 | -1.25 | 3.84 | -1.94 | 9.76 |
| 50%~75% | 39.37 | 37.62 | 1.75 | 3.64 | -3.17 | 10.73 |
| **75%~100%** | **56.19** | **39.28** | **16.91** | **5.94** | **0.16** | **11.16** |

CBT vs. COMB

| Predicted Benefit Strata | GLL | | | Random Features | | |
| --- | --- | --- | --- | --- | --- | --- |
|  | treated with CBT | treated with COMB | est. benefit | est. benefit (permuted data) | | |
|  | mean | mean | mean | mean | 5th percentile | 95th percentile |
| 0~25% | 35.75 | 30.71 | 5.04 | 7.70 | 2.00 | 14.00 |
| 25%~50% | 36.86 | 34.79 | 2.07 | 7.52 | 2.11 | 12.60 |
| **50%~75%** | **39.28** | **30.83** | **8.44** | **7.55** | **1.47** | **12.58** |
| **75%~100%** | **55.79** | **36.81** | **18.98** | **9.11** | **4.03** | **14.34** |

FLX vs. COMB

| Predicted Benefit Strata | GLL | | | Random Features | | |
| --- | --- | --- | --- | --- | --- | --- |
|  | treated with FLX | treated with COMB | est. benefit | est. benefit (permuted data) | | |
|  | mean | mean | mean | mean | 5th percentile | 95th percentile |
| 0~25% | 39.2 | 35.6 | 3.6 | 3.13 | -2.33 | 8.26 |
| 25%~50% | 34.5 | 33.0 | 1.6 | 4.17 | -0.7 | 9.29 |
| 50%~75% | 36.1 | 32.0 | 4.1 | 3.5 | -1.8 | 8.89 |
| 75%~100% | 38.4 | 34.0 | 4.4 | 2.61 | -2.03 | 7.39 |

III. Patient CDRS-R Benefits Stratified by Model Prediction

Supplemental Figure 1: This is a graphical depiction of table 3 in the main text.

The mean and standard deviation (error bar) of observed 12 W CDRS-R score for subjects that are in different predicted benefit strata. Panel 1A-C show CBT vs FLX, CBT vs COMB, and FLX vs COMB respectively. For CBT vs. FLX, the participants that are predicted to benefit the most from FLX (75%~100% strata) showed significant benefit from FLX with on average 16.9 CDRS-R difference at 12 week. For CBT vs. COMB the participants that are predicted to benefit the most from COMB (50%~75% and 75%~100% strata) showed significant benefit from COMB with significant CDRS-R difference at 12 week. * indicates statistically significant difference *p*<0.05 after adjusting for multiple comparison.

1A.


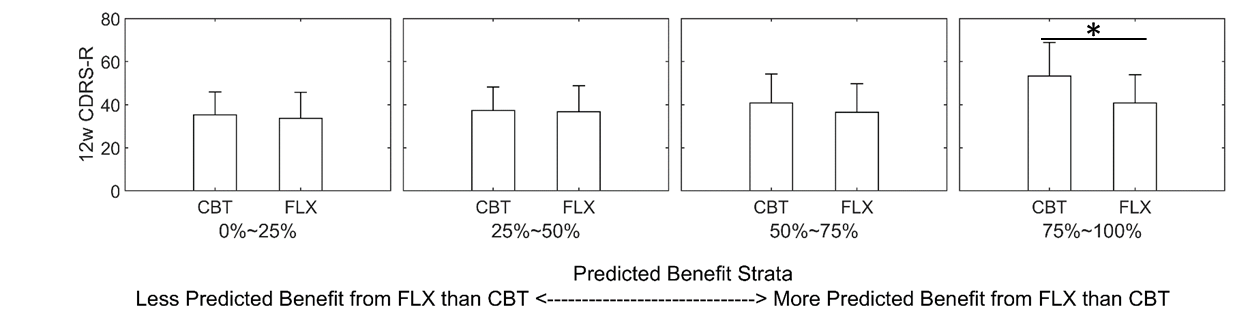


1B.


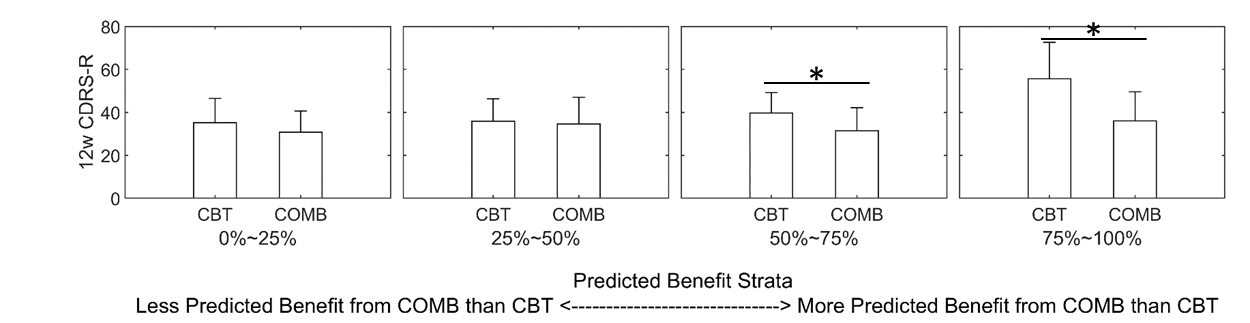


1C.


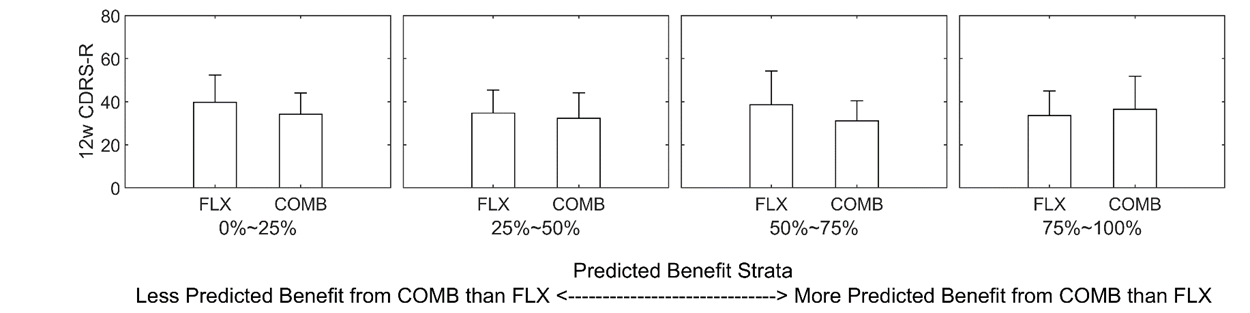


IV. Translation of model into a clinical algorithm

Supplemental Figure 2: Proposed method of implementing a treatment selection algorithm:


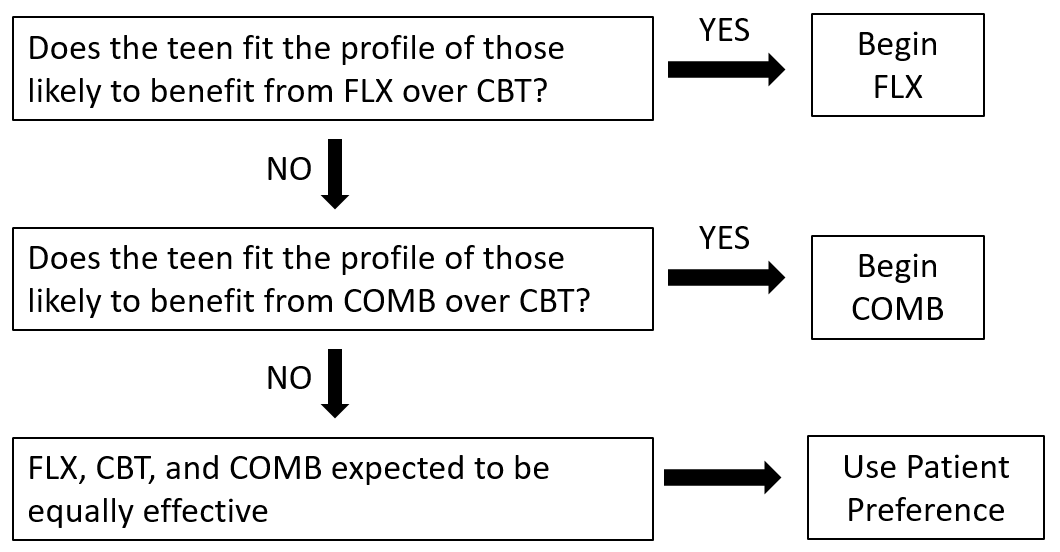


V. Predictors and Moderators Identified in Prior Studies

Supplemental Table 4:

In this table we listed variables that were identified as predictors or moderators in other studies (column 1 and 2), and explain why these variables were not identified as predictors (column 3) or moderators (column 4-6). NC indicates that a variable is not significantly correlated with CDRS-R at 12 weeks. The variables that are not correlated with CDRS-R at 12 weeks considering all patients without considering their treatment group (column 3) are not eligible predictors in the present study. The variables that are not correlated with CDRS-R at 12 weeks considering patients in specific treatment groups (column 4-6) are not eligible moderators in the present study for that specific treatment. For variables that are significantly correlated with CDRS-R at 12 weeks, we list the variable in our final model that renders it conditionally independent with CDRS-R at 12 weeks. For example, anxs_tot significantly correlates with CDRS-R at 12 weeks, but the correlation vanishes given baseline CDRS-R. In other word, anxs_tot does not contain additional information regarding CDRS-R at 12 weeks given baseline CDRS-R. Therefore, anxs_tot is not in our final model since information in anxs_tot regarding CDRS-R at 12 weeks is captured in CDRS-R at baseline. Similarly, for canxiedo is correlated with CDRS-R at 12 weeks in the CBT group, therefore it is eligible as a moderator for CBT, however, canxiedo and CDRS-R at 12 weeks is rendered conditionally independent by baseline CDRS-R, i.e. considering baseline CDRS-R, the information in canxiedo regarding 12 week CDRS-R in CBT group becomes irrelevant.

| **Predictors/Moderators Previously Reported in Literature** | | **Predictor** | **Moderator** | | |
| --- | --- | --- | --- | --- | --- |
| **Variable Description** | **Variable Name** |  | **CBT** | **FLX** | **COMB** |
| BDI | bdi_tot | NC | NC | NC | NC |
| BHS | bhs_tot | CDRS-R | CDRS-R | NC | NC |
| Cognitive Distortion | cogdist | CDRS-R | CDRS-R | NC | NC |
| SIQ Total | siqtot | CDRS-R | CDRS-R | NC | Attn Probs |
| Anxiety (BSI) | anxiety | NC | NC | NC | NC |
| MASC Total Score | anxs_tot | CDRS-R | NC | NC | NC |
| Current Anxiety Disorders | canxiedo | NC | CDRS-R | NC | NC |
| Any co-morbidity currently present | anycomrb | NC | CDRS-R | NC | NC |
| Physical Abuse | pabuse | NC | NC | NC | NC |
| Sexual Abuse | sabuse | NC | CDRS-R | NC | NC |
| adolescent report of mother behavior | cbqmbeh | NC | NC | NC | NC |
| adolescent report of mother-adolescent dyadic behavior | cbqmdyad | NC | NC | NC | NC |
| parent report of adolescent behavior | cbqpbeh | NC | NC | NC | NC |
| parent report of parent-adolescent dyadic behavior | cbqpdyad | NC | NC | NC | NC |
| Family Assessment Measure t value | t_values | NC | NC | NC | NC |
| Race White | race | NC | NC | NC | NC |
| Age | age_c | NC | NC | NC | NC |
| Income | income5 | NC | NC | NC | NC |
| Suicidal Ideation | ctaly1a9 | NC | NC | NC | NC |
